# P-903. Human Parechovirus Central Nervous System (CNS) Infection in Infants: A Multicenter Retrospective Case Series of the 2022 outbreak

**DOI:** 10.1093/ofid/ofae631.1094

**Published:** 2025-01-29

**Authors:** Avni Sheth, Nisha Shah, Oliver Fortin, Christina Gagliardo, Sarmistha Bhaduri Hauger, Sarah J Calardo, Marina Santos-Oren, Cristina Tomatis Souverbielle, Guliz Erdem, Pablo J Sanchez, Adil Solaiman, Nada Harik, Sarah B Mulkey, Rachel Downey, Dimitrios Angelis, Emily Ansusinha, Lina Chalak, Susan Russo, Paul Teran, Aspasia Katragkou

**Affiliations:** ATLANTIC HEALTH SYSTEM, Morristown, New Jersey; University of Kansas School of Medicine-Wichita, Wichita, Kansas; Children's National Hospital, Washington DC, District of Columbia; Atlantic Health System/Thomas Jefferson University, Morristown, New Jersey; Dell Children's Medical Center; Dell Medical School at the University of Texas at Austin, Austin, Texas; Nemours Children's Health Delaware, Wilmington, Delaware; University of Texas-Southwestern, Dallas, Texas; Nationwide Children's Hospital, columbus, Ohio; Nationwide Children's Hospital, columbus, Ohio; Nationwide Children's Hospital - The Ohio State University, Columbus, OH; Nemours Children's Health, Wilmington, Delaware; Children’s National Hospital/ GWU School of Medicine , Washington, DC; Children's National Hospital/ George Washington University School of Medicine and Health Sciences, Washington, District of Columbia; Dell Children's Medical Center of Central Texas, Austin, Texas; University of Texas-Southwestern, Dallas, Texas; Children's National Hospital, Washington DC, District of Columbia; University of Texas-Southwestern, Dallas, Texas; Dell Children's Medical Center, Austin, TX; University of Kansas School of Medicine-Wichita, Wichita, Kansas; Goryeb Children's Hospital, Atlantic Health System, Morristown, New Jersey

## Abstract

**Background:**

Human parechovirus (PeVA) typically causes self-limiting respiratory and gastrointestinal infections. However, in infants, it can cause severe sepsis-like illness and CNS disease. In 2022, a CDC Health Advisory reported severe PeVA infections in young infants in multiple states. Our aim was to describe the clinical features, laboratory characteristics, and outcomes in infants with PeVA CNS infection during the 2022 multistate outbreak.

**Methods:**

A multicenter retrospective chart review was conducted at 7 children’s hospitals (6 states) of infants with PeVA CNS infection. Cases were identified by positive CSF PCR testing using the FilmArray meningitis/encephalitis panel (BioFire Diagnostics, Salt Lake City, UT). Data were entered in REDCap and pertinent demographic and clinical data were analyzed.

**Results:**

103 infants were admitted from March-October 2022 with 30 (29%) of the admissions occurring in July. Patients’ median age was 18 days, 55% were male, and 93% born full term. The median length of hospital stay was 3.3 days. 19% required admission to an intensive care unit, 29% had increased oxygen requirement, and 5% required inotropic support. The most common clinical manifestations were fever (84%), irritability (63%), poor feeding (37%), tachycardia (31%), seizures (23%) and hypothermia (19%). The median peripheral white blood cell count was 5.6 x10^9^/L and median CSF white blood cell count was 3 cells/mm^3^. The median CRP was 0.3 mg/dL and 54% of the infants had transaminitis. Brain MRIs were obtained in 55 cases; 31 (56%) demonstrated abnormalities with diffusion restriction involving white matter and thalami. EEGs were obtained in 40 cases; 22 (55%) revealed abnormalities. 99% of our infants survived, 13% were discharged with anti-seizure medications, and 10% received developmental follow-up.

**Conclusion:**

PeVA should be considered in young infants presenting with fever or hypothermia, sepsis-like symptoms, or meningitis even with no laboratory findings of significant inflammatory responses. PeVA CNS infection in young infants can cause neurologic injury requiring long term neurodevelopmental follow up. Nationwide epidemiological studies are necessary to elucidate the dynamics of this infection.

**Disclosures:**

**Cristina Tomatis Souverbielle, MD**, Merck, inc: Grant/Research Support

